# Training in eight low-and middle-income countries: lessons learned from a pilot study using the WHO-TDR dissemination and implementation massive open online course

**DOI:** 10.3389/frhs.2023.1217619

**Published:** 2024-01-19

**Authors:** Ashlin Rakhra, Cole Hooley, Meredith P. Fort, Mary Beth Weber, LeShawndra Price, Hoa L. Nguyen, Manuel Ramirez, Adamson S. Muula, Mina Hosseinipour, Kingsley Apusiga, Victor Davila-Roman, Joyce Gyamfi, Kezia Gladys Amaning Adjei, Josephine Andesia, Annette Fitzpatrick, Pascal Launois, Ana A. Baumann

**Affiliations:** ^1^ Department of Population Health, NYU Grossman School of Medicine, New York City, NY, United States; ^2^ School of Social Work, Brigham Young University, Provo, UT, United States; ^3^ Colorado School of Public Health, University of Colorado, Aurora, CO, United States; ^4^ Rollins School of Public Health, Emory University, Atlanta, GA, United States; ^5^ National Institute of Allergy and Infectious Diseases, National Institutes of Health, Bethesda, MD, United States; ^6^ Epidemiology, University of Massachusetts Medical School, Worcester, MA, United States; ^7^ Center for the Prevention of Chronic Diseases -CIIPEC, Institute of Nutrition of Central America and Panama (INCAP), Guatemala City, Guatemala; ^8^ College of Medicine, University of Malawi, Zomba, Malawi; ^9^ Department of Medicine, University of North Carolina at Chapel Hill, Chapel Hill, NC, United States; ^10^ Department of Physiology, Kwame Nkrumah University of Science & Technology, Kumasi, Ghana; ^11^ School of Medicine, Washington University in St. Louis, St. Louis, MO, United States; ^12^ Global Health Program, New York University Global College of Public Health, New York City, NY, United States; ^13^ Academic Model Providing Access to Healthcare (AMPATH), Moi University, Eldoret, Kenya; ^14^ School of Public Health, University of Washington, Seattle, WA, United States; ^15^ Research Capacity Strengthening (RCS) Unit, Special Programme for Research and Training in Tropical Diseases (TDR), WHO, Geneva, Switzerland; ^16^ Division of Public Health Sciences, Department of Surgery, Washington University in St. Louis, St. Louis, MO, United States

**Keywords:** implementation research, dissemination & implementation research, capacity building, massive open online course (MOOC), non-communicable chronic diseases

## Abstract

**Introduction:**

Non-communicable diseases (NCDs) are a leading cause of morbidity and mortality in low-and middle- income countries (LMICs). Despite this, a lack of funding, training and mentorship for NCD investigators in LMICs exists. In an effort to gain knowledge and skills to address these gaps, participants from the Global Research on Implementation and Translation Science (GRIT), a consortium of studies in eight LMICs and their networks, attended the dissemination and implementation (D&I) massive open online course (MOOC) developed by the Special Programme for Research and Training in Tropical Diseases at the World Health Organization to strengthen D&I capacity building. Here, we report on the pilot of this MOOC, which was implemented during the SARS COVID-19 pandemic from April- November 2020.

**Methods:**

Participants completed pre-and post-training questionnaires to assess self-reported D&I competencies, general research skills, and research mentor access and quality. D&I competencies were measured by use of a scale developed for a US-based training program, with change in competency scores assessed by paired t test. We used univariate statistics to analyze the data for all other outcomes.

**Results:**

Of the 247 participants enrolled, 32 (13%) completed all course requirements, 21 (9%) completed the pre-and post-surveys and are included in the analysis. D&I competency scores suggest improvement for those who had complete pre- and post-assessments. Trainee's average score on the full competency scale improved 1.45 points (0–5 scale) from pre- to post-test; all four subscales also showed evidence of improvements. There were small but not significant increases in competencies for grant writing, proposal/ manuscript writing and presentations from pre- to post-test assessment. 40% of trainees reported access to a research mentor and 12% reported access to a D&I specific mentor. Participants reported barriers (e.g., unstable internet access and challenges due to COVID-19) and facilitators (e.g., topical interests, collaboration with colleagues) to completing the MOOC.

**Conclusions:**

Although COVID-19 affected program usage and completion, the MOOC was feasible. We also had signals of effectiveness, meaning among LMIC participants completing the course, there was improvement in self-report D&I competency scores. Recommendations for future D&I trainings in LMICs include (1) adding more topic specific modules (i.e., NCD research, general research skills) for scalability; (2) fostering more collaboration with participants across LMICs; and (3) establishing partnerships with D&I mentors for course participants.

## Introduction

Non-communicable diseases (NCDs) are the leading cause of mortality worldwide that disproportionately impact low and middle-income countries (LMICs) (1). With 80% of deaths from NCDs occurring in LMICs, the role of local research capacity and relevant research informing policy and practice is crucial (2). Despite this, there has been a particular lack of funding, training and mentorship for NCD investigators in LMICs (3).

The Special Programme for Research and Training in Tropical Diseases (TDR) at the World Health Organization (WHO) developed the Massive Open Online Course (MOOC), which aims to disseminate implementation research concepts (4). The primary goal of the course is to strengthen capacity building and improve training opportunities, targeting local public health researchers, practitioners and policy-makers (4). The course delivers dissemination and implementation (D&I) research education in LMICs where access to formal learning pathways, such as university courses in implementation research, may be limited (5). Investing in research capacity and training in LMICs reduces disease burden by building local research capacity and ensuring that those who are being trained are best equipped to address the needs of their communities (6–9).

MOOCs have steadily gained popularity given the accessibility, affordability, and effectiveness of the courses (4, 10, 11). The TDR MOOC on Implementation Research (IR) has shown to improve participant knowledge and understanding of implementation research and increased participants' ability to apply the course concepts to professional practice (12). While this MOOC was developed with a focus on infectious diseases of poverty, the course concepts can be applied to strengthening implementation research capacity for NCDs and other disciplines (12, 13).

The goals of this paper are to describe the pilot evaluation outcomes of one of the 2020 MOOC-D&I trainings conducted by the Global Research on Implementation and Translation Science (GRIT) consortium as part of the GRIT's ongoing mentorship and capacity building programs, and share barriers, facilitators, and recommendations to enhance future D&I training opportunities in LMICs.

## Methods

### TDR MOOC on IR

The TDR MOOC on IR is a step-by-step online training for public health researchers and decision-makers that focuses on design and implementation of research projects (12). Core concepts of implementation research are addressed in five modules including: (1) identifying the challenges of various health settings; (2) assessing the appropriateness of existing disease control strategies; (3) developing new interventions and strategies by working with communities and stakeholders; (4) specifying implementation research questions; and (5) designing rigorous research projects, including how to identify implementation outcomes, evaluating effectiveness, and making plans to scale-up implementation in real life settings (12). The course includes homework assignments, the requirement of completing and passing at least four quizzes and a final assignment with a peer-review component. The five modules were open to participants from May 11, 2020, to Sept. 25, 2020. The final exam was available to take after the five modules were completed until October 23, 2020, and participants were required to complete the peer-review assessment by November 6, 2020. Participants completed an electronic survey at the beginning and conclusion of the MOOC to evaluate the change in knowledge and self-assessed competencies. Subsequently, participants were asked to share barriers and facilitators to completing the MOOC. Participants who completed all course components received a MOOC certificate of completion.

### Participants

There were two sets of participants in this study. The first were participants from the Global Research on Implementation and Translation Science (GRIT) Consortium funded by the National Heart Lung and Blood Institute. The consortium consists of research teams from eight countries, five of which (Guatemala, Ghana, Kenya, India, and Vietnam) test implementation strategies to deliver evidence-based interventions within these countries for the prevention, treatment, and control of hypertension and three of which (Malawi, Nepal, and Rwanda) provide capacity building in NCD and D&I research needed to close the gap between research and practice (3, 6). Specifically, all countries have partnership between D&I mentors and hypertension physicians in the U.S. and in country. Members from all countries were invited to GRIT workshops about implementation science and hypertension care, and all countries have developed formal and informal infrastructures of mentoring in D&I and research in general (14). The MOOC was an added structure in which consortium members decided to engage to support enhancing D&I knowledge for GRIT members.

The invitation to participate in the MOOC was open to all consortium members. Additionally, GRIT participants were encouraged to share the announcement with their respective networks. The second set of participants were not part of the GRIT Consortium and were recruited through snowball sampling through the GRIT network. We did not have inclusion or exclusion criteria. Our recruitment email invited anyone interested in the MOOC with a brief description of the course, timeline and expectations. Enrollment was open from April 6 to May 5, 2020 and the course ran from May 11, 2020 to November 6, 2020.

### Measures

This was a pilot study aimed to see if training D&I via an online platform was feasible across eight LMICs. The primary outcome of this study was competency in D&I research. Surveys were distributed via Qualtrics (15). We also examined four secondary outcomes including: (1) D&I mentor access and quality; (2) general research mentor access and quality; (3) general research skill competencies, in manuscript writing, proposal writing, making scholarly presentations, and grant writing; and (4) a qualitative assessment of barriers and facilitators to completing the MOOC. While the TDR MOOC does not have a formal mentorship as part of the course, GRIT members are connected formally or informally with their D&I members in either delivering interventions or enhancing capacity building in D&I and hypertension care. Additionally, unique to this training was the expectation that results from the MOOC training could be used as potential future research ideas as part of GRIT capacity building efforts.

The current study is a single-group, pre-post study design to assess changes in D&I research competencies, measure mentor access and quality, and describe general research skills among participants in the TDR MOOC. Additionally, barriers and facilitators to completing the course were examined. Researchers originally developed the competency measure for D&I trainings in the United States (5, 8, 16). Others have subsequently used this measure to assess D&I competencies for the WHO MOOC internationally (10, 17). The 43 item self-report measure is organized into four subscales: (1) definitions, background, and rationale, (2) theory and approaches, (3) design and analysis, and (4) practice-based considerations (5, 8) using a 5-point Likert scale (i.e., Not at all to Extremely).

A secondary outcome of this study was D&I mentor access and quality, measured through three questions added to the original survey. The first question asked trainees whether they had access to a D&I mentor (answer options: yes, no, not sure). If the trainee had a D&I mentor, they were asked two follow-up questions. The first follow-up question assessed the quality of the mentoring (“how would you rate the overall quality of the mentoring you received from your D&I mentor?”). Trainees answered using a 7-point Likert scale with anchoring verbiage at 3 points (1- very low, 7-very high). The second follow-up question assessed the degree to which the D&I mentoring met their expectations: “to what extent do you feel your D&I mentor is meeting your expectations?” Trainees answered using a 7-point Likert scale with anchoring verbiage at 3 points (1-Not at all, 7-Completely).

A third outcome examined the general research mentor access and quality, with three additional survey questions. The first research mentor question asked trainees whether they had access to a research mentor (answer options: yes, no, not sure). Trainees with a research mentor were asked a follow-up question about mentoring quality and the degree to which the mentoring met their expectations. The same questions, with answer options, that were asked to assess the quality of the mentorship and met expectations for their general research mentor were asked for those with a D&I specific mentor.

A fourth outcome measured general research skill competencies, in manuscript writing, proposal writing, making scholarly presentations, and grant writing. Trainees rated their level of competency for each of these items using a 5-point Likert scale (1 not at all to 5 extremely).

The final outcome examined was barriers and facilitators to completing the MOOC. Trainees who completed the MOOC were asked: “what enabled you to complete the MOOC?” Trainees who completed some but not all of the MOOC were asked: “what enabled you to complete some of but not all the MOOC components?” Trainees who did not complete all of the MOOC were asked: “what prevented you from being able to complete the MOOC?” All trainees were asked: “What changes/support would help future participants complete the MOOC?” The questions pertaining to barriers and facilitators were open-ended and included in the post-assessment survey.

MOOC participant demographic information was also collected. Specifically, participants provided their gender, age, education, country, work position, work location, and GRIT participation.

### Analysis

Quantitative analyses were conducted in Stata 16.1. A paired t-test was used to determine if trainees' D&I competencies and general research competencies changed from pre- to post-test. The trainees' average total D&I competency score and their average scores for each sub-scale were calculated (8, 10). The analytic sample for our primary outcome only included trainees with complete pre- and post- D&I competency measures; those with missing data were excluded. Chi-square tests, Fisher exact tests, and independent two sample *t*-tests using demographic variables were obtained to determine if the trainees without complete D&I competency measures differed from those with complete pre- and post-measures. Some respondents had missing demographic variables and could not be included in the comparison assessment. Demographic variable tests were run separately; the lowest number of missing variables was 5 and the highest was 9.

The results from that analysis suggest that there were no meaningful differences between those with complete D&I competency scores and those without. As such, only the results for trainees with complete pre- and post-test D&I competency measures are reported. Similarly, the analytic sample for the fourth outcome measuring general research competency only included respondents with complete pre- and post-data for that measure. There were more respondents with complete pre- and post-data for general research competency compared to the D&I competency completers. The same diagnostic tests were run on the general research competency completers and non-completers. No meaningful difference was found between the two groups.

A univariate statistic was used to analyze the data for the second and third outcomes. Data for those outcomes came from the pre-test survey data. Observations with missing data were removed. Finally, the qualitative data for the final outcome, barriers and facilitators, were analyzed using thematic analysis to identify themes, patterns and areas of overlap in participant's open ended survey responses (18).

## Results

### MOOC participation

247 individuals from the GRIT Consortium and ancillary networks enrolled in the MOOC; 116 (47%) completed the pre-assessment survey, 101 (41%) attempted any quizzes, 59 (24%) completed all quizzes, 35 (14%) completed the final exam, and 32 participants (13%) completed all course requirements, but only 21 (8%) completed both pre and post-surveys and therefore these are the ones included in the analysis.

Table 1 outlines the demographic characteristics of the trainees who completed both the pre- and post-competency measures (*n* = 21) and the demographics for all participants that initially enrolled in the MOOC (*n* = 116). Most of the trainees with complete pre-and post-D&I competency measures were female (57%); had a master's degrees (76%); were from Rwanda (33%) and Malawi (24%); were not GRIT Consortium members (62%); did not have previous D&I training experience (67%); and the average participant age was 35 ± 4.5.

**Table 1:** Demographic characteristics of trainees.

	With complete pre/post D&I competency (*n *= 21) *n* (%)		At least one item in pre-test survey (*n *= 116) *n* (%)	
Gender				
Male	9	(43%)	61	(53%)
Female	12	(57%)	49	(42%)
Other	0	(0%)	1	(1%)
Missing	0	(0%)	5	(4%)
Education				
PhD/MD	2	(10%)	18	(16%)
Master's degree	16	(76%)	61	(53%)
Some graduate school	0	(0%)	4	(3%)
Bachelor's degree	3	(14%)	27	(23%)
Some college	0	(0%)	1	(1%)
Missing	0	(0%)	5	(4%)
Country				
Ghana	3	(14%)	9	(8%)
Guatemala	0	(0%)	4	(3%)
India	0	(0%)	4	(3%)
Kenya	0	(0%)	10	(9%)
Malawi	5	(24%)	22	(19%)
Nepal	4	(19%)	10	(9%)
Rwanda	7	(33%)	45	(39%)
Vietnam	2	(10%)	7	(6%)
Missing	0	(0%)	5	(4%)
Work position^a^				
Academic	7	(33%)	36	(31%)
Clinician	2	(10%)	10	(9%)
Leadership	0	(0%)	17	(15%)
Research (other)	6	(29%)	27	(23%)
Multiple positions	3	(14%)	9	(8%)
Other	2	(10%)	8	(7%)
Missing	1	(5%)	9	(8%)
Work location				
Ministry of health	3	(14%)	14	(12%)
Research center	5	(24%)	25	(22%)
University	12	(57%)	54	(47%)
WHO	1	(5%)	1	(1%)
Community health center	0	(0%)	3	(3%)
Hospital	3	(14%)	28	(24%)
Other	0	(0%)	8	(7%)
Missing	1	(5%)	9	(8%)
GRIT participants				
TREIN	6	(29%)	16	(14%)
HyTREC	2	(10%)	19	(16%)
Not part of GRIT	13	(62%)	74	(64%)
Missing	0	(0%)	7	(6%)
Previous D&I training				
Yes	7	(33%)	38	(33%)
No	14	(67%)	73	(63%)
Missing	0	(0%)	5	(4%)
Age, m ± sd (range)	35 ± 4.5 (25–42)		35 ± 5.8 (25–63)^b^	

^a^ Values sum to more than 100% because respondents could select multiple work locations.

^b^ Sample size for the age was (*n* = 111).

### D&I competencies

The self-reported D&I competency scores improved for those with completed pre- and post-competency measures (see Table 2). Trainees' average score improved on the full scale by 1.45 points., the, The *definitions, background, and rationale subscale* improved by 1.36 points. The *theory and approaches subscale* improved by 1.63 points. The *design and analysis subscale* improved by 1.45 points. *practice-based considerations* subscale improved by 1.37 points.

**Table 2:** D&I competencies pre- to post test change*.*

D&I research competency areas (1 = not at all, 5 = extremely, *n* = 21)	Pre-test Mean ± SD	Post-test Mean ± SD	95% CI mean difference
Full scale	2.12 ± 0.93	3.57 ± 0.97	1.04–1.86*
Definitions, background, and rationale	2.54 ± 0.93	3.90 ± 0.94	0.93–1.79*
Theory and approaches	2.01 ± 0.98	3.64 ± 1.06	1.08–2.17*
Design and analysis	1.94 ± 0.95	3.39 ± 1.02	1.07–1.83*
Practice-based considerations	2.08 ± 1.02	3.45 ± 0.94	0.97–1.78*
General research competency areas (1 = not at all, 5 = extremely, *n* = 33)			
Manuscript writing	3.48 ± 0.97	3.48 ± 1.03	−0.34–0.34
Proposal writing	3.42 ± 0.90	3.45 ± 1.12	−0.30–0.36
Scholarly presentations	3.58 ± 1.17	3.76 ± 1.12	−0.19–0.55
Grant writing	2.24 ± 1.03	2.42 ± 1.12	−0.13–0.49

* *p* < .001.

Participants reported that access to D&I mentoring was low (not reported in Table). Only 12% (*n* = 14) of the MOOC trainees who completed the pre-survey (*n* = 116) indicated that they had a D&I mentor. Of those that had a D&I mentor, 63% reported the quality of mentoring was above average, with 21% rating the quality as “very high.” 71% reported a 4 or 5 out of 7 regarding that their mentor met their expectations for mentorship, where 4 reflected moderately meeting expectations.

Access to general research mentoring was reported by the participants as being higher compared to D&I mentoring. Forty percent (*n* = 46) of the MOOC trainees that completed the pre-survey reported having a research mentor. Around 52% of those with a research mentor rated the mentoring quality as above average. Similar to the D&I mentoring, 46% reported a 4 or 5 out of 7 reflecting that the mentor met expectations for mentorship, where 4 represented moderately meeting expectations.

Research competency scores among those with complete pre- and post-survey responses (*n* = 33) varied. While the scores generally improved from pre- to post-survey, the differences were not statistically meaningful. Participant manuscript writing scores remained constant between pre- and post-test [3.48 ± 0.97 vs. 3.48 ± 1.03, *t*(32) = 0.00, *p *> 0.05]. Proposal writing improved slightly from [3.42 ± 0.90 vs. 3.45 ± 1.12, *t*(32) = 0.19, *p* > .05] Scholarly presentation scores improved 0.18 points (3.58 ± 1.17 vs. 3.76 ± 1.12, *t*(32) = 1.00, *p* > 0.05. Grant writing scores improved also increased 0.18 points [2.24 ± 1.03 vs. 2.42 ± 1.12, *t*(32) = 1.18, *p* > 0.05].

### Barriers and facilitators

Participants reported major barriers preventing them from completing the course including lack of time, other work commitments or additional responsibilities placed on them due to the COVID-19 pandemic, and lack of stable and consistent internet connection. Participants identified time management skills, an interest in the topics addressed by the course, and recognizing the opportunity to learn as driving factors in completing the course. Additional facilitators included collaborating with other participants, supervisors and colleagues; the course flexibility (i.e., pre-recorded sessions to adapt to participant's schedule as opposed to live sessions); and increased time to work on the course due to personal or professional changes during the COVID-19 pandemic.

### Course recommendations

Participant recommendations for future MOOC sessions included: (1) greater mentorship from the GRIT stakeholders throughout the course; (2) greater collaboration among participants across LMICs; (3) having the ability to retake modules or quizzes for greater understanding of a specific topic; (4) incorporating an NCD module or more NCD related examples; (5) minimizing website navigation challenges; (6) facilitating access to a reliable internet connection; and (7) more course flexibility. To enhance flexibility, participants suggested having a flexible deadline for the peer assessment, having all modules accessible at the beginning of the course with a final deadline, and having extra time for assignments.

## Discussion

This study examined the experience of participants from eight LMICs in one of the 2020 TDR MOOCs on IR. Using the data from the pre-and post-assessment surveys, the self-reported D&I competencies were analyzed as well as barriers and facilitators to completing the course, which provide recommendations and implications for future MOOCs. Although there was a low retention rate in the MOOC, participants completing the post survey showed improvements in their D&I competencies. Participants reported low access to D&I mentors, limited access to general research mentors, and low self-reported competency for manuscript and scientific writing.

Existing literature on previous MOOCs have generally shown lower completion rates (10, 19–23), including a systematic review reporting the majority of MOOCs in the study reported completion rates of less than 10% (23). The course completion rate in this MOOC (13%) was likely impacted by a couple of variables. First, internet access was a major barrier for retention in this study, which has been shown in similar studies (10). MOOCs, by design, enroll large groups of students, including both active and passive participants. Reconceptualizing retention to only include participants who substantively engaged with the course might provide a more accurate picture of program metrics (19). Third, the timeline in which we started the MOOC was challenging. Enrollment took place in April 2020 with a course start date in May 2020, right before several of the participating countries started the lock down to prevent further spread of the SARS-CoV-2 virus. Fourth, as soon as the cohort started the training, the MOOC website moved to be hosted by another company and the transition posed some issues with access to the videos. With the larger movement towards online courses and trainings, future guardrails to develop and maintain websites for online learning will be important (20–22).

Due to the global uncertainty of the COVID-19 pandemic, coupled with TDR platform issues, the first module was extended three months until the end of July 2020. The remaining modules adhered to the original timelines, with a spacing of two weeks between each module. During this period, 53% of enrolled participants no longer engaged in course activities. When asked about the barriers for participation in the post-assessment survey, participants shared that the lack of stable internet, other work commitments and responsibilities, and needing more time to complete the course were key barriers that affected their participation in this course. These barriers have been reported by participants from previous MOOCs (10, 13, 17), but they were likely intensified by the COVID-19 pandemic and associated lockdowns.

This was a pilot study aiming to see if we could provide capacity building in D&I using a MOOC platform in LMICs. The results indicate strong evidence of improvement with self-reported D&I competencies similar to previous courses (10). The subscale that had the largest change was *theory and approaches* and the subscale with the least change was *definitions, background and rationale* and *practice-based considerations*. These results differ from other D&I trainings where a majority of participants reported the largest change in the *definitions, background, and rationale* subscale (8, 10). The difference in results may be related to the composition of participants, where 33% of participants in this study had previous D&I training before the course. The general research competency scores in manuscript writing, proposal writing, and giving scholarly presentations did not change in a meaningful way from pre-to-post test. These findings suggest that general research capacities, not specific to D&I, should be targeted by future capacity-building activities, particularly grant writing. Accordingly, the TDR WHO has developed a flexible and interactive D&I toolkit to support capacity building and proposal writing (24).

The need for increased mentoring and guidance was a prominent theme in the recommendations submitted by the participants, as only 14% reported having a mentor in D&I research. Even though every country has a D&I consultant, the limited access to D&I mentors may be a reflection of very few researchers being trained in the emerging field of implementation science in LMICs. Evaluation of other D&I trainings in high resource settings have shown the importance of networking and mentoring, as well as time, for the development of academic outcomes (8, 25), and previous MOOCs with added support beyond mentorship (i.e., meetings for participants to discuss modules, Q&A sessions, discussion forums) demonstrated an increase in participant engagement (10, 26). Future D&I capacity trainings in LMICs should include greater mentorship and support throughout the course as it could contribute to higher course completion and improved overall D&I competency reporting (7, 27). It is also worth noting that many participants were not a part of the GRIT Consortium, but rather recruited and enrolled through collaboration with GRIT Consortium members, demonstrating that MOOCs are an effective tool to extend trainings beyond existing consortiums and partnerships.

### Limitations

The major limitation of this paper is the small number of participants that completed the course. Additionally, evaluation data was comprised of only self-report data and, therefore subjected to bias and social desirability. Lastly, we did not follow up with participants to ask whether they were able to apply what they learned. The lack of opportunity to practice what they learned has been a challenge described by participants in previous MOOCs (25, 28).

### Future directions and implications

Despite the challenges and limitations, partnering with the Special Programme for Research and Training in TDR MOOC is a feasible and scalable strategy to increase D&I training in LMICs. The use of D&I competency metrics allows for further evaluation on how to design training in D&I. In the future, research partners may add specific modules, such as hypertension care and D&I grant writing. Some of this is already being done as part of capacity building initiatives (17, 29). In moving forward, setting up and strengthening a collaborative practice whereby mentoring and peer collaboration across countries could be beneficial to all in enhancing the capacity for D&I research training (29).

## Conclusions

This was a pilot study, and as such, the main hypothesis was to see if we could foster D&I capacity building in LMICs using the TDR MOOC platform. Using pre-post surveys, augmented by the analysis of the open questions from the trainees, this study follows similar designs of other capacity building efforts and adds to the literature in capacity building in D&I in LMIC, showing that self-report D&I competencies improved after the training.

## Data Availability

The raw data supporting the conclusions of this article will be made available by the authors, without undue reservation.
